# Novel use of culturomics to identify the microbiota in hospital sink drains with and without persistent VIM-positive *Pseudomonas aeruginosa*

**DOI:** 10.1038/s41598-020-73650-8

**Published:** 2020-10-13

**Authors:** Jannette Pirzadian, Susan P. Harteveld, Shanice N. Ramdutt, Willem J. B. van Wamel, Corné H. W. Klaassen, Margreet C. Vos, Juliëtte A. Severin

**Affiliations:** Department of Medical Microbiology and Infectious Diseases, Erasmus MC University Medical Center Rotterdam, Rotterdam, The Netherlands

**Keywords:** Applied microbiology, Clinical microbiology, Microbiome

## Abstract

In hospitals, Verona Integron-encoded Metallo-beta-lactamase (VIM)-positive *Pseudomonas aeruginosa* may colonize sink drains, and from there, be transmitted to patients. These hidden reservoirs are difficult to eradicate since *P. aeruginosa* forms biofilms that resist disinfection. However, little is known on the composition of these biofilms. Therefore, culturomics was used for the first time to investigate the viable microbiota in four hospital sink drain samples with longstanding VIM-positive *P. aeruginosa* drain reservoirs (inhabited by high-risk clone, sequence type ST111), and four drain samples where VIM-positive *P. aeruginosa* was not present. Microbial load and composition varied between samples, yielding between 471–18,904 distinct colonies and 8–20 genera. In two VIM-positive drain samples, *P. aeruginosa* was the most abundantly-isolated microorganism, and found in combination with other Gram-negative bacteria, *Citrobacter*, *Enterobacter*, or *Stenotrophomonas*. *P. aeruginosa* was in low abundance in the other two VIM-positive samples, and found with Gram-positive cocci (*Enterococcus* and *Staphylococcus*) or *Sphingomonas*. In VIM-negative drain samples, high abundances of Gram-negative non-fermenting bacteria, including *Acinetobacter*, non-aeruginosa *Pseudomonas* spp., *Acidovorax*, *Chryseobacterium*, *Flavobacterium*, and *Sphingobium*, as well as *Candida*, were cultured. Although additional experiments are needed to draw more firm conclusions on which microorganisms enable or inhibit VIM-positive *P. aeruginosa* persistence, our data provide unique insights into the microbial compositions of sink drain inlets.

## Introduction

Hospital plumbing systems provide a significant breeding ground for bacteria. Numerous studies have reported isolating clinically-relevant and opportunistic Gram-negative bacteria from hospital sinks and drain water^1^. Multidrug-resistant (MDR) *Pseudomonas aeruginosa* has been extensively described in these sites, often in direct patient surroundings^1,2^. *P. aeruginosa* is well-known for forming biofilms, which hinder disinfection, and allow this bacterium to persist in hospital sink drains or siphons for prolonged periods of time^3,4^. Therefore, when carbapenemase-producing, MDR *P. aeruginosa* emerged in hospitals, possible environmental reservoirs and transmission pathways garnered greater focus. Within our tertiary care hospital, Verona Integron-encoded Metallo-beta-lactamase (VIM)-positive *P. aeruginosa* has been detected at low endemicity in patients since 2003. As part of our prevention efforts, environmental sampling was implemented in 2011, which revealed that sink drains have been acting as persistent environmental sources for these bacteria^5,6^. Through multilocus sequence typing, we determined that within our hospital, the majority of patient- and environment-derived VIM-positive *P. aeruginosa* isolates were sequence type ST111, a globally-distributed, high-risk clone responsible for extensively drug-resistant nosocomial infections^7,8^. Conversely, environmental sampling also showed that in some sink drains within our hospital, VIM-positive *P. aeruginosa* has never been cultured before.

During sink use, particles or droplets carrying *P. aeruginosa* can disperse from sink drains, contaminating the surrounding sink environment and objects used for patient care^9,10^; in this way, reservoirs become an environmental source of transmission for hospital-acquired infections. Recent reports have shown that the design of sinks and the location of drains contribute to the dispersal of these resistant bacteria into the surrounding environment. However, it is currently unknown why VIM-positive *P. aeruginosa* forms reservoirs and persists in some hospital sink drains, but not in others. We hypothesize that differences in drain microbiota play a role. Antagonism and competition between *P. aeruginosa* and other microorganisms have been previously documented^11^, but these interactions have never yet been described in sink environments.

Sink drain microbiota has been until now unexplored, since in these niches, only MDR bacteria are surveilled. To investigate microbiota, molecular- or culturomics-based methods are used. Culturomics is a technique that uses a diverse repertoire of culture conditions to determine microbial composition^12^. Numerous culture conditions are used to enable growth of minority populations, limit overgrowth by majority populations, and provide insight on the interactions microorganisms experience in a given niche. Culture conditions may also be customized to emulate the environment of origin for difficult-to-culture species, increasing the likelihood of isolation in culture^13^. Compared to molecular methods, culturomics is better able to detect microorganisms in low loads, but detection is limited to viable, cultivable species^12^; however, viable species, especially pathogens, are risks to patients when present in their immediate environment.

Despite its unique advantages, culturomics has never yet been used to study the microbial burden of the innate hospital environment. Therefore, we employed culturomics for the unique application of detecting the viable microbiota in eight separate drain samples from our hospital. We examined four sink drains persistently colonized by VIM-positive *P. aeruginosa*, and four sink drains where VIM-positive *P. aeruginosa* has never been detected before, even after exposure to culture-positive patients. Our aim was to detect majority and minority microbial populations from each sample, and, more specifically, to characterize the microbiota in drains where VIM-positive *P. aeruginosa* has been either successful or unsuccessful at establishing longstanding reservoirs. By broadening our focus from MDR bacteria to all existing and viable microorganisms, we were able to identify microorganisms that potentially enable or inhibit colonization by VIM-positive *P. aeruginosa*. This knowledge may contribute to the development of environmental probiotic treatments to decolonize future sink drains from these notoriously persistent bacteria, or prevent future reservoir formation.

## Methods

### Setting

The Erasmus MC University Medical Center is a tertiary care hospital in Rotterdam, the Netherlands. Adult intensive care units consisted of single-patient rooms, some with anterooms. The general surgery and gastrointestinal surgery wards consisted of two- and four-bed patient rooms. Environmental sampling for VIM-positive *P. aeruginosa* occurred sporadically before 2011, and systematically after 2011 following increasing numbers of VIM-positive *P. aeruginosa* patients. Indications for environmental sampling were (1) unexpected colonization of a patient in either the room or ward, and (2) follow-up after an environmental intervention, such as when drain plugs were installed in the sinks from six wards (J. A. Severin, unpublished data). Until patient wards closed in May 2018 for demolition, VIM-positive *P. aeruginosa* was frequently cultured from the environment, including from the positive sink drains chosen for this study.

As this study did not involve humans, animals, or interventions, in accordance with Dutch law, neither ethical approval nor waiving of ethical approval was required. Furthermore, all methods described were carried out under relevant guidelines and regulations.

### Sink selection and sample collection

Previous environmental sampling data were used to select sinks. Only sinks from wards most affected by VIM-positive *P. aeruginosa* colonization were considered for inclusion. Four positive sinks (represented by ^+^) were chosen that were considered hotspots for VIM-positive *P. aeruginosa* over a period of at least four years (Supplementary Fig. S1). Four negative sinks (represented by ^−^) were chosen where VIM-positive *P. aeruginosa* had not been cultured over a period of four years, even after exposure to use by or waste from admitted, culture-positive patients (Supplementary Fig. S1). On average, 1.5 environmental cultures were taken per sink per year. Immediately prior to each culturomics experiment, the sink environment was sampled again: the wash basin, counter, faucet aerator, and drain inlet were swabbed (BBL CultureSwab Plus, BD Diagnostics, Sparks, MD, United States). Swabs were incubated overnight at 35 °C in 5 ml of a CAZ-VAN enrichment broth consisting of tryptic soy broth with 2 mg/l ceftazidime (CAZ) and 50 mg/l vancomycin (VAN). From this broth, DNA was extracted using MagNa Pure 96 (Roche Diagnostics, Almere, the Netherlands) according to the manufacturer’s instructions. DNA was then screened for *bla*_VIM_ in-house using real-time PCR: 5 µl DNA were added to 5 µl primer/probe mix [forward primer 5′-GCAAATTGGACTTCCYGTAA-3′, reverse primers 5′-GACGGTGATGCGTACGTTG-3′ and 5′-CCCTAAGGGCATCAACTCC-3′, probe 5′-Cy5-TTTCATGACGACCGCGTCGG-3′-BHQ-2 (Eurogentec, Maastricht, the Netherlands)] and 10 µl LightCycler 480 Probes Master (Roche Diagnostics, Almere, the Netherlands). PCR was performed in a LightCycler 480 Real-Time PCR Instrument (Roche Diagnostics, Almere, the Netherlands) using the following program: pre-denaturation (95 °C/5 min), followed by 50 cycles of denaturation (95 °C/5 s) and annealing (60 °C/30 s). VIM-positive *P. aeruginosa* strain S04 90 was used as a positive control^14^.

**Figure 1:**
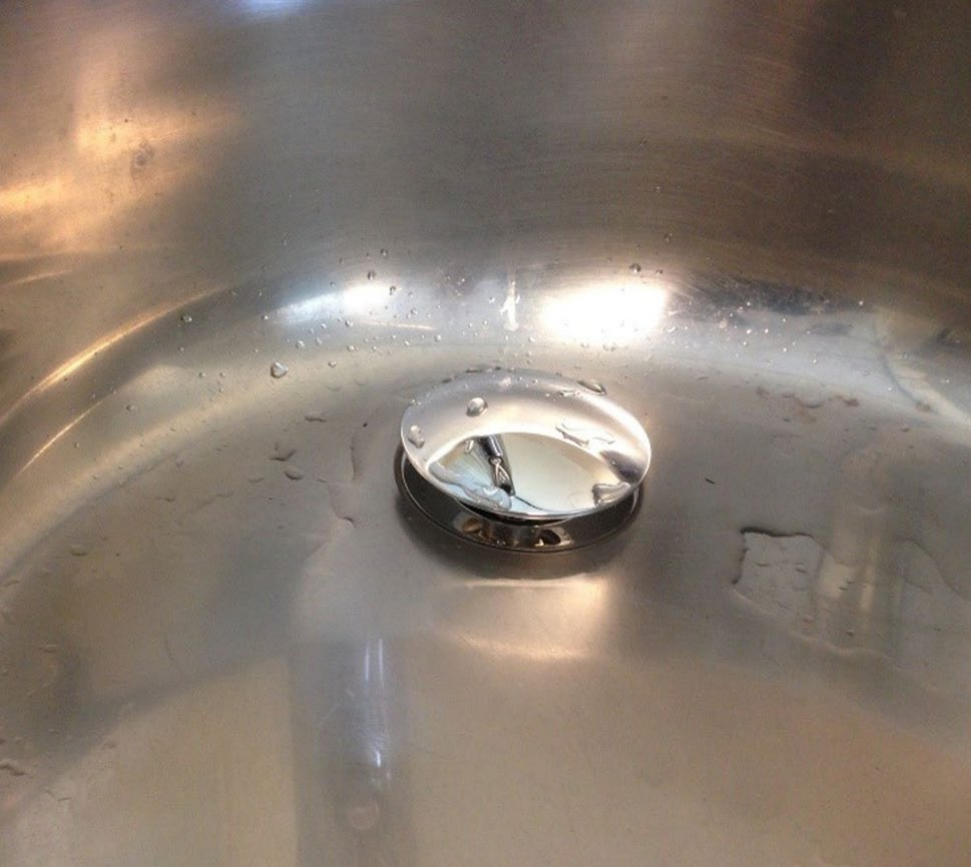
Drain plugs used in this study. Only sinks containing identical stainless steel drain plugs were considered for inclusion. The surface of these plugs contained no holes, and when screwed into the drain, completely covered the drain inlet.

Drain plugs (article no. UD.526.51, Raminex, Utrecht, the Netherlands) from the eight selected sinks were aseptically removed and stored in separate 1.1 l sterile sonication containers. Drain plugs were entirely comprised of stainless steel, and were all of identical size and design (Fig. 1).

### Culturomics

Drain samples were obtained by sonicating drain plugs to dislodge biofilms. We hypothesized that drain plugs were contaminated by the same microorganisms colonizing the upper ends of siphons and drain inlets, and that these microorganisms were most at risk of dispersal from sink use. Before sonicating, the underside of each drain plug was scraped with a sterile scalpel to sample the topmost layer of biofilm. The scalpel was consecutively pressed onto four Tryptic Soy Agar II with 5% Sheep Blood (TSA) plates (BD Diagnostics, Breda, the Netherlands). Each of the four plates was incubated under a different condition: one at 35 °C in aerobic conditions, one at 35 °C in aerobic conditions with 5% CO_2_, one at 35 °C in microaerophilic conditions, and one at 35 °C in anaerobic conditions (Table 1, Supplementary Table S2).

**Table 1:** Overview of culturomics culture media and conditions.

Culture medium	Room temperature			35 °C			
		0.45	TS		0.45	TS	CO_2_
**Aerobic conditions (n = 87)**							
Blood culture enrichment	✕	✕	✕	✕	✕	✕	
Brain heart infusion agar + 10 µg/ml kanamycin	✕			✕			✕
Brain heart infusion agar + 10 µg/ml vancomycin	✕			✕			✕
Brain heart infusion broth + 10 mg/l vitamin B6	✕			✕			✕
Brucella agar + 5% sheep blood	✕			✕			✕
*Burkholderia cepacia* selective agar	✕			✕			✕
Cetrimide agar	✕			✕			✕
Chocolate agar	✕			✕			✕
Eosin methylene blue agar	✕			✕			✕
ESBL agar	✕			✕			✕
GC agar + supplement VX + 2% hemoglobin	✕			✕			✕
Hektoen enteric agar	✕			✕			✕
*Legionella* buffered charcoal yeast extract agar + 0.4 g/l L-cysteine hydrochloride	✕			✕			
*Legionella* buffered charcoal yeast extract agar + antibiotics	✕			✕			
MacConkey agar	✕			✕			✕
Middlebrook 7H10 agar	✕			✕			✕
Mueller Hinton agar + 10 µg/ml kanamycin	✕			✕			✕
Mueller Hinton agar + 10 µg/ml vancomycin	✕			✕			✕
Mueller Hinton agar + 5% sheep blood	✕			✕			✕
Mueller Hinton agar + hospital tap water	✕			✕			✕
Phenyl mannitol agar + 75 g/l sodium chloride	✕			✕			✕
Reasoner's 2A agar	✕			✕			✕
Tryptic soy agar + 5% sheep blood	✕	✕	✕	✕	✕	✕	✕
Tryptic soy broth + 2 mg/l ceftazidime, 50 mg/l vancomycin (CAZ-VAN enrichment broth)	✕			✕			✕
Tryptic soy broth + 4 mg/l gentamicin, 1 mg/l norfloxacin	✕			✕			✕
Tryptic soy broth + 4 mg/l tobramycin, 1 mg/l norfloxacin	✕			✕			✕
Tryptic soy agar + 5% sheep blood, scalpel (no sonication)				✕			✕
Tryptic soy agar + 5% sheep blood (5 cumulative min sonication)				✕			
Brucella agar + 5% sheep blood (5 cumulative min sonication)				✕			
**Microaerophilic conditions (n = 47)**							
Brain heart infusion agar + 10 µg/ml kanamycin	✕			✕			
Brain heart infusion agar + 10 µg/ml vancomycin	✕			✕			
Brucella agar + 5% sheep blood	✕			✕			
*Burkholderia cepacia* selective agar	✕			✕			
Cetrimide agar	✕			✕			
Chocolate agar	✕			✕			
Eosin methylene blue agar	✕			✕			
ESBL agar	✕			✕			
GC agar + supplement VX + 2% hemoglobin	✕			✕			
Hektoen enteric agar	✕			✕			
*Legionella* buffered charcoal yeast extract agar + 0.4 g/l L-cysteine hydrochloride	✕			✕			
*Legionella* buffered charcoal yeast extract agar + antibiotics	✕			✕			
MacConkey agar	✕			✕			
Middlebrook 7H10 agar	✕			✕			
Mueller Hinton agar + 10 µg/ml kanamycin	✕			✕			
Mueller Hinton agar + 10 µg/ml vancomycin	✕			✕			
Mueller Hinton agar + 5% sheep blood	✕			✕			
Mueller Hinton agar + hospital tap water	✕			✕			
Phenyl mannitol agar + 75 g/l sodium chloride	✕			✕			
Reasoner’s 2A agar	✕			✕			
Tryptic soy agar + 5% sheep blood	✕	✕	✕	✕	✕	✕	
Tryptic soy agar + 5% sheep blood, scalpel (no sonication)				✕			
**Anaerobic conditions (n = 45)**							
Bacteroides bile esculin agar	✕			✕			
Blood culture enrichment	✕	✕	✕	✕	✕	✕	
Brain heart infusion agar + 10 µg/ml kanamycin	✕			✕			
Brain heart infusion agar + 10 µg/ml vancomycin	✕			✕			
Brucella agar + 5% sheep blood	✕	✕	✕	✕	✕	✕	
CDC anaerobe 5% sheep blood agar + phenylethyl alcohol	✕			✕			
Cetrimide agar	✕			✕			
Chocolate agar	✕			✕			
ESBL agar	✕			✕			
MacConkey agar	✕			✕			
Mueller Hinton agar + 10 µg/ml kanamycin	✕			✕			
Mueller Hinton agar + 10 µg/ml vancomycin	✕			✕			
Mueller Hinton agar + 5% sheep blood	✕			✕			
Mueller Hinton agar + hospital tap water	✕			✕			
Tryptic soy agar + 5% sheep blood	✕	✕	✕	✕	✕	✕	
Tryptic soy agar + 5% sheep blood, scalpel (no sonication)				✕			
Tryptic soy agar + 5% sheep blood (5 cumulative min sonication)				✕			
Brucella agar + 5% sheep blood (5 cumulative min sonication)				✕			

In the table, a cross mark means that the indicated condition was tested for that culture medium. 0.45 = sonication fluid was subjected to 0.45 µm-sized pore prefiltration prior to inoculation. TS = sonication fluid was subjected to thermic shock pretreatment prior to inoculation. CO_2_ = media were incubated at 35 °C in aerobic conditions with 5% CO_2_.

*CDC* Centers for Disease Control and Prevention; *ESBL* extended spectrum beta-lactamase; *GC* Gonococcal; *CAZ* ceftazidime; *VAN* vancomycin.

Subsequently, drain plugs were subjected to sonication using the protocol for orthopedic prostheses to diagnose prosthesis-associated infections: the drain plug was covered in 115 ml 0.9% NaCl, scraped, shaken rigorously for 30 s inside the sonication container, and sonicated for 1 min at 40 kHz, 800 W at 100% power in an ultrasonic water bath (Bandelin Electronic, Berlin, Germany)^15^. Following sonication, the container was shaken rigorously again for 30 s. In a laminar flow cabinet, 100 µl of sonication fluid were inoculated into 5 ml of each broth, and onto every agar plate using disposable spreaders; 3 ml of sonication fluid were inoculated into each blood culture bottle (BD BACTEC, BD Diagnostics, Breda, the Netherlands) with sterile syringes and needles. Culturomics experiments were independently performed for each drain sample.

Culture media were chosen from a repertoire of routine and specialized diagnostic culture media, because the likelihood of cultivating human commensal flora and pathogens was high (Table 1, Supplementary Table S2). Several conditions used were inspired by the seminal culturomics article by Lagier *et al*.: active filtration of sonication fluid through 0.45 µm-pore-sized filters was performed prior to inoculation to isolate smaller cell sizes; thermic shock pretreatment was performed by submerging sonication fluid in an 80 °C water bath for 20 min prior to inoculation to isolate existing spores; different atmospheric conditions were used to differentiate by oxygen requirement; blood culture enrichment followed by subculture on TSA was used to stimulate the growth of clinical isolates; antibiotic-supplemented media were used to isolate MDR species; eosin methylene blue agar was used to isolate enteric microorganisms; Reasoner’s 2A agar was used to isolate water-associated microorganisms; and cetrimide agar was used to select for *Pseudomonas* spp. (Table 1, Supplementary Table S2)^12^. In addition to incubation at 35 °C, media were also incubated at room temperature to mimic sink environment temperatures (Table 1, Supplementary Table S2). Finally, a custom agar medium was created by our laboratory: Mueller Hinton II Agar (BD Diagnostics, Breda, the Netherlands) was made according to the manufacturer’s instructions, but purified water was replaced with tap water from the tested wards (Table 1, Supplementary Table S2).

Then, the drain plug was aseptically transferred to another sterile sonication container with 115 ml 0.9% NaCl, scraped, shaken for 30 s, and sonicated for an additional 4 min (5 cumulative min sonication time) to retrieve viable microorganisms potentially present in deeper layers of biofilm that were not dislodged during the initial sonication step^16,17^. This container was shaken again for 30 s, and 100 µl were respectively inoculated onto one TSA and one Brucella agar plate (BD Diagnostics, Breda, the Netherlands) that were incubated aerobically at 35 °C, and onto one TSA and one Brucella agar plate that were incubated anaerobically at 35 °C (Table 1, Supplementary Table S2). In total, 179 culture conditions were tested per drain sample (Table 1, Supplementary Table S2). All commercially-prepared culture media were provided by BD Diagnostics, Breda, the Netherlands or Thermo Fisher Scientific, Bleiswijk, the Netherlands. For culture media created in-house, materials were supplied by BD Diagnostics, Breda, the Netherlands or Sigma-Aldrich Chemie, Zwijndrecht, the Netherlands.

### Growth characterization and quantification

Cultures were checked regularly for growth, and incubated for a total of 6 weeks or until nearly confluent. Morphologically-unique colonies that were visible to the unaided eye were subcultured onto TSA or onto the medium on which they initially grew. Rapid identification of isolates was performed using MALDI-TOF MS (Bruker Daltonik, Bremen, Germany) according to the manufacturer’s instructions. Genus identification was accepted for scores above 1.80, and species identification was accepted for scores above 2.0. In case MALDI-TOF MS could not identify a microorganism to the genus level, identification was performed by partial 16S rRNA gene sequencing.

To calculate relative abundances per drain sample, total colony counts were enumerated for each genus. Growth in broths and blood culture bottles was uncountable and not included; growth in these conditions was only used to document the presence/absence of microorganisms not found in solid media.

From each drain sample, a selection of *P. aeruginosa* isolates and other Gram-negative bacteria were screened for *bla*_VIM_ using PCR as described above. In case few Gram-negative bacteria were found, all Gram-negative isolates were then screened for *bla*_VIM_.

### 16S rRNA gene sequencing

To identify microorganisms not identifiable by MALDI-TOF MS, DNA was isolated as described above, and sequenced targeting the first ~ 550 bp of the 16S rRNA gene. For isolates that could not be subcultured from original cultures, DNA was extracted by picking a colony from an original culture using a toothpick, and suspending the inoculum in 25 µl water and 5 µl proteinase K before heating (56 °C/10 min followed by 99 °C/5 s). Briefly, 10 µl DNA were added to 2.5 µl 10 × primer mix [5 µM each of forward primer 5′-AGAGTTTGATCMTGGYTCAG-3′ and reverse primer 5′-CTTTACGCCCARTRAWTCCG-3′ (Eurogentec, Maastricht, the Netherlands)] and 12.5 µl FastStart PCR Master (Roche Diagnostics, Almere, the Netherlands). PCR was performed in a thermocycler using the following program: pre-denaturation (95 °C/5 min), followed by 20 cycles of denaturation (94 °C/30 s), annealing (55 °C/30 s), and extension (72 °C/1 min). Prior to sequencing, 20 µl of PCR product were treated with ExoSAP-IT *Express* PCR Product Cleanup Reagent (Thermo Fisher Scientific, Bleiswijk, the Netherlands) using the recommended conditions. Sequencing was performed by BaseClear, Leiden, the Netherlands. A standard BLAST analysis was performed using the obtained DNA sequences to identify the microorganisms involved.

### *Legionella* and *Acanthamoeba* PCRs

To detect the presence of *Legionella* spp. or *Acanthamoeba* spp. in drain samples, 10 ml from both 1- and 5-min sonication fluids were centrifuged at 4000 rpm/15 min to pellet cells, and the supernatants were decanted. Cells were resuspended in residual sonication fluid to make concentrated suspensions, and then DNA extraction was performed as described above. For *Acanthamoeba* PCR, a bead beating step (30 s at a frequency of 30 r/s) preceded DNA extraction. For the detection of *Legionella* spp., an in-house real-time PCR was performed based on the assay described by Templeton *et al*. with minor modifications^18^; *L. pneumophila* CCUG 33,152 was used as a positive control. For the detection of *Acanthamoeba* spp., an in-house real-time PCR was performed based on the assay described by Qvarnstrom *et al*. with minor modifications^19^; *A. castellanii* CCAP 1501/1B was used as a positive control.

## Results

### Sink selection

Among positive sinks that were selected based on persistent colonization by VIM-positive *P. aeruginosa*, three were from patient rooms in adult intensive care units (Drains A^+^, C^+^, and D^+^), while the fourth sink was from a patient room in the gastrointestinal surgery ward (Drain B^+^) (Supplementary Fig. S1). PCR on swab cultures immediately prior to each culturomics experiment confirmed the presence of VIM-positive *P. aeruginosa* in these sinks’ drains.

Among negative sinks that were selected based on never having cultured VIM-positive *P. aeruginosa* in the preceding four-year period, one was from a patient room anteroom in adult intensive care (Drain E^−^), one from a patient room in the general surgery ward (Drain F^−^), one from a communal patient bathroom in the gastrointestinal surgery ward (Drain G^−^), and one from a dirty utility room for healthcare workers in the general surgery ward (Drain H^−^) (Supplementary Fig. S1). PCR on swab cultures immediately prior to each culturomics experiment confirmed the absence of VIM-positive *P. aeruginosa* in these sinks’ drains. The anteroom with a negative sink (Drain E^−^) adjoined a patient room with a positive sink (Drain C^+^); additionally, the wastewater pipelines of these sinks were connected.

### Culturomics

**Figure 2:**
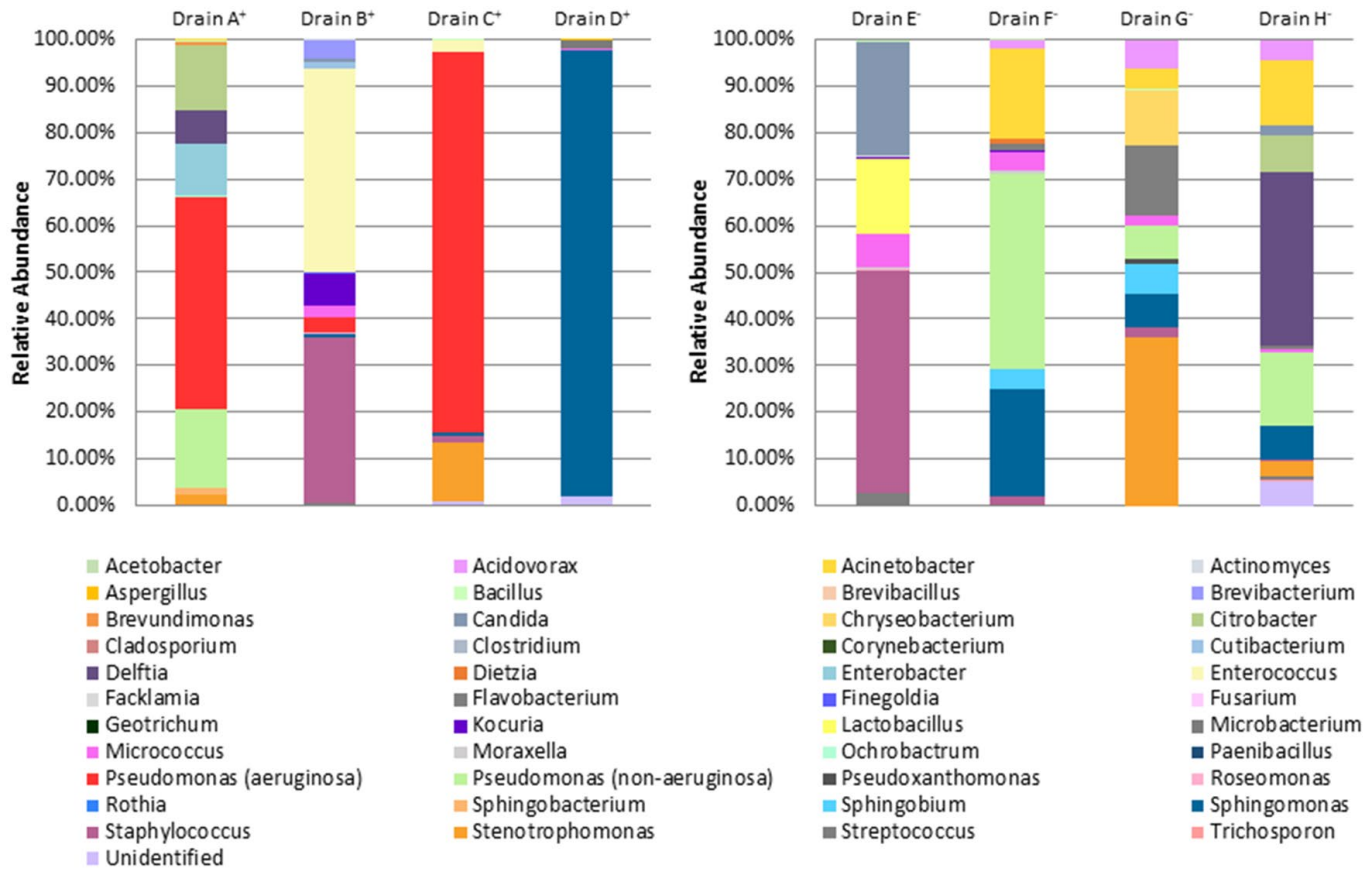
Relative abundance of all genera. Drains A^+^, B^+^, C^+^, and D^+^ were sink drain samples containing VIM-positive *P. aeruginosa*. Drains E^−^, F^−^, G^−^, and H^−^ were sink drain samples that did not contain VIM-positive *P. aeruginosa*. Relative abundances are shown as percentages.

Culturomics was performed on drain samples obtained from the eight selected sinks. Results are presented in Fig. 2 and Table 2, while a list of identifiable species is presented in Supplementary Table S3. In the four positive sink drain samples, VIM-positive *P. aeruginosa* was successfully cultured using culturomics, but the relative bacterial load differed; in two samples (Drains A^+^ and C^+^), VIM-positive *P. aeruginosa* was the most abundantly-isolated microorganism, accounting for 46% and 81% of all colonies, respectively. From Drain A^+^, we also observed moderate relative abundances of *Citrobacter* (14%), *Delftia* (7%), *Enterobacter* (11%), and *Pseudomonas putida* group isolates (17%), specifically *P. putida* and *P. monteilii* (Supplementary Table S3). From Drain C^+^, only *Stenotrophomonas* was in moderate relative abundance (13%), while other genera appeared suppressed due to the overrepresentation of *P. aeruginosa*. Fewer than 1% of colonies from Drain C^+^ were unidentifiable (Table 2). In the two other positive sink drain samples (Drains B^+^ and D^+^), VIM-positive *P. aeruginosa* was cultured, but in lower relative abundance compared to other genera. Drain B^+^ primarily grew *Enterococcus* (44%) and coagulase-negative staphylococci (36%), while *P. aeruginosa* was only detected among 3% of colonies. Drain D^+^ was dominated by growth of *Sphingomonas* (96%), while *P. aeruginosa* was found among < 0.1% of colonies. Two percent of colonies from Drain D^+^ were unidentifiable (Table 2).

**Table 2:**
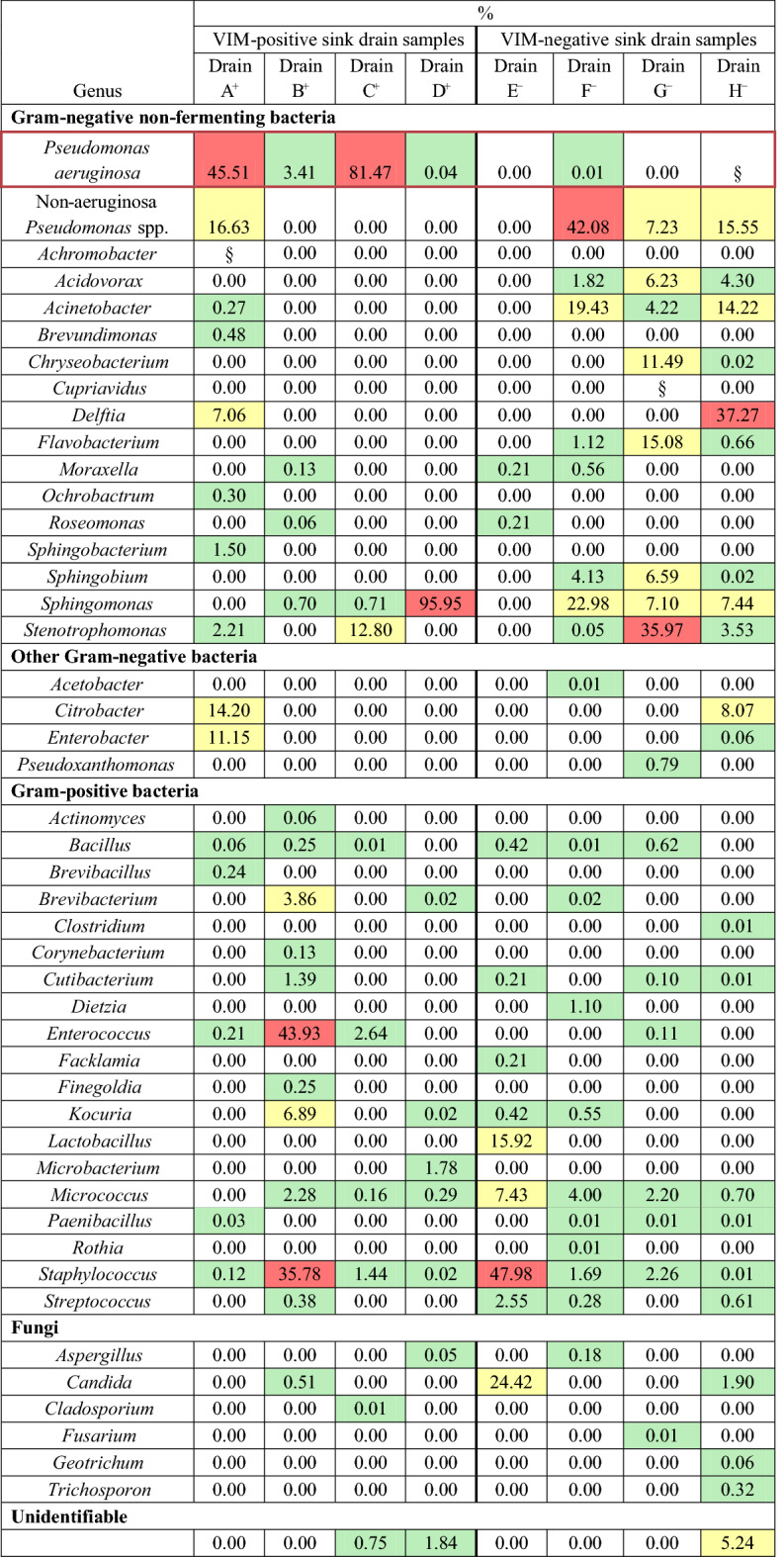
Growth results of drain samples.

Drains A^+^, B^+^, C^+^, and D^+^ were sink drain samples containing VIM-positive *P. aeruginosa*. Drains E^−^, F^−^, G^−^, and H^−^ were sink drain samples that did not contain VIM-positive *P. aeruginosa*. Relative abundances are shown as percentages, and color-coded for low abundance (green; < 5%), moderate abundance (yellow; 5–30%), and high abundance (red; > 30%); abundances of 0.00% (shown in white) indicate a complete absence of that genus in a sample. Genera are grouped in the categories Gram-positive or Gram-negative bacteria, fungi, and unidentifiable. A large amount of non-fermenting Gram-negative bacteria was discovered, so these are grouped separately.

§ = *Achromobacter* (Drain A^+^), *Cupriavidus* (Drain G^−^), and VIM-negative *P. aeruginosa* (Drain H^−^) grew in liquid culture media, and were uncountable.

In two out of four negative sink drain samples, VIM-negative *P. aeruginosa* isolates were found (Drains F^−^ and H^−^), but no other *P. aeruginosa* was cultured (Table 2). Drain E^−^ grew moderate relative abundances of *Candida* (24%), *Lactobacillus* (16%), and *Micrococcus* (7%), and a high relative abundance of coagulase-negative staphylococci (48% of colonies). This drain was physically near and closely connected to Drain C^+^, but the drain microbiota between these samples showed major differences: only *Bacillus*, *Micrococcus*, and *Staphylococcus* were present in both (Table 2). Drain F^−^ grew moderate relative abundances of *Acinetobacter* (19%) and *Sphingomonas* (23%), and a high relative abundance of *Pseudomonas fluorescens* group isolates (42% of colonies), specifically *P. gessardii* and *P. proteolytica* (Supplementary Table S3). Drain G^−^ grew moderate relative abundances of *Acidovorax* (6%), *Chryseobacterium* (11%), *Flavobacterium* (15%), *Sphingobium* (7%), *Sphingomonas* (7%), and *P. aeruginosa* group isolates (7%), specifically *P. oleovorans* and *P. pseudoalcaligenes* (Supplementary Table S3); a high relative abundance of *Stenotrophomonas* (36% of colonies) was also recorded. Drain H^−^ grew moderate relative abundances of *Acinetobacter* (14%), *Citrobacter* (8%), *Sphingomonas* (7%), and *P. fluorescens* group isolates (16%), specifically *P. proteolytica*, *P. brenneri*, *P. rhodesiae*, and *P. koreensis* (Supplementary Table S3); a high relative abundance of *Delftia* (37% of colonies) was also recorded. Five percent of colonies from Drain H^−^ were unidentifiable (Table 2). Except for Drains B^+^ and E^−^, sink drain samples were populated by few Gram-positive bacteria and fungi (Fig. 2).

Microbial load and genus composition varied widely between samples, yielding between 471 and 18,904 distinct colonies, and between 8 and 20 identifiable genera. There was a small number of colonies that could not be subcultured from original cultures, suggesting that these microorganisms depended on polymicrobial co-culture for survival or were very sensitive to certain culture conditions. In total, 44 genera were identified across all samples, and most could be identified to species level using MALDI-TOF MS (Supplementary Table S3). However, 16S rRNA sequencing was necessary to identify the following to genus or species level: *Cladosporium* spp. (for Drain C^+^); *Brevibacterium* spp. and *Microbacterium* spp. (for Drain D^+^); *Acetobacter indonesiensis*, *Flavobacterium tructae*, *Moraxella osloensis*, and *Sphingobium yanoikuyae* (for Drain F^−^); *Bacillus circulans*, *Cupriavidus* spp., *Pseudoxanthomonas mexicana*, *Sphingobium yanoikuyae*, and *Sphingomonas aerolata* (for Drain G^−^); and *Chryseobacterium* spp., *Chryseobacterium ureilyticum*, and *Clostridium algidixylanolyticum* (for Drain H^−^).

Growth in broths and blood culture bottles was uncountable, and only used to document the presence/absence of microorganisms not found in solid media. The only genera that were exclusively cultivated in these conditions and not in solid culture media were *Achromobacter* and *Cupriavidus* (Table 2).

Furthermore, three culture conditions specialized in *Pseudomonas* selection were compared. Across all samples, cetrimide agar grew all non-aeruginosa *Pseudomonas* spp., except in Drain G^−^; however, *P. aeruginosa* was only detected in two out of four positive sink drain samples (Drains A^+^ and C^+^) using this medium (Table 3). The CAZ-VAN enrichment broth (used to culture swabs for *bla*_VIM_ PCR) grew all non-aeruginosa *Pseudomonas* spp., except in Drains G^−^ and H^−^; VIM-positive *P. aeruginosa* was detected in three out of four positive sink drain samples (Drains A^+^, B^+^, and C^+^) using this medium (Table 3). Finally, Mueller Hinton (MH) agar made with tap water from the tested wards grew several water-associated microorganisms, including non-aeruginosa *Pseudomonas* spp. from all samples; VIM-positive *P. aeruginosa* from all four of the positive sink drain samples was also detected using this medium (Table 3).

**Table 3:** Growth in *Pseudomonas* selective media.

Drain sample	Genus	Cetrimide agar	CAZ-VAN enrichment broth	Mueller Hinton agar with hospital tap water
Drain A^+^	*Acinetobacter*			✕
	*Brevundimonas*			✕
	*Citrobacter*		✕	✕
	*Delftia*			✕
	*Enterobacter*			✕
	*Pseudomonas aeruginosa*	✕	✕	✕
	*Pseudomonas putida* group	✕	✕	✕
	*Stenotrophomonas*			✕
Drain B^+^	*Bacillus*			✕
	*Enterococcus*			✕
	*Kocuria*			✕
	*Micrococcus*			✕
	*Pseudomonas aeruginosa*		✕	✕
	*Roseomonas*			✕
	*Staphylococcus*			✕
Drain C^+^	*Enterococcus*			✕
	*Pseudomonas aeruginosa*	✕	✕	✕
Drain D^+^	*Aspergillus*			✕
	*Kocuria*			✕
	*Microbacterium*			✕
	*Micrococcus*			✕
	*Pseudomonas aeruginosa*			✕
	*Sphingomonas*			✕
Drain E^−^	*Candida*		✕	✕
	*Lactobacillus*			✕
	*Micrococcus*			✕
	*Staphylococcus*			✕
Drain F^−^	*Acidovorax*			✕
	*Acinetobacter*			✕
	*Micrococcus*			✕
	*Pseudomonas fluorescens* group	✕	✕	✕
	*Sphingomonas*			✕
	*Stenotrophomonas*			✕
Drain G^−^	*Acidovorax*			✕
	*Acinetobacter*			✕
	*Chryseobacterium*			✕
	*Flavobacterium*		✕	✕
	*Pseudomonas aeruginosa* group			✕
	*Sphingobium*			✕
	*Stenotrophomonas*			✕
Drain H^−^	*Acidovorax*			✕
	*Acinetobacter*			✕
	*Candida*		✕	
	*Citrobacter*		✕	✕
	*Delftia*			✕
	*Micrococcus*			✕
	*Pseudomonas fluorescens* group	✕		✕
	*Sphingomonas*			✕
	*Stenotrophomonas*			✕
	*Streptococcus*			✕
	*Trichosporon*			✕

Drains A^+^, B^+^, C^+^, and D^+^ were sink drain samples containing VIM-positive *P. aeruginosa*. Drains E^−^, F^−^, G^−^, and H^−^ were sink drain samples that did not contain VIM-positive *P. aeruginosa*. In the table, a cross mark means that growth was observed in the indicated condition for that genus.

*CAZ* ceftazidime; *VAN* vancomycin.

### *bla*_VIM_ in other Gram-negative isolates

One hundred eighty-eight Gram-negative bacterial isolates, including *P. aeruginosa*, were screened for *bla*_VIM_ using PCR. Besides *P. aeruginosa* from positive sink drain samples, *P. putida* group isolates from Drain A^+^ were also positive for *bla*_VIM_. All other Gram-negative bacterial isolates were negative.

### *Legionella* and *Acanthamoeba* in drain samples

Aliquots from each 1- and 5-min sonication fluid suspension were screened for *Legionella* spp. and *Acanthamoeba* spp. using PCR, but all samples were negative.

## Discussion

In this study, a microbial culturomics approach was applied to eight hospital sink drain samples to determine the viable microbiota in drains with and without persistent VIM-positive *P. aeruginosa*. In these niches, cultures are often targeted toward MDR bacteria following an outbreak, and not toward all available microorganisms; the novelty of our study is that all cultivable microorganisms were examined. Moreover, to the best of our knowledge, culturomics has never been used to investigate the innate hospital environment before. Our data provide unique insights into the microbial compositions of sink drains and inlets. These sites are worrisome transmission sources because they are more accessible to the environment outside of the sink drain, and are coincidentally microbial hotspots from which MDR bacteria may disperse. Furthermore, *P. aeruginosa* colonization in siphons has mainly been reported at distal ends rather than within water distribution systems^11^, so targeting *P. aeruginosa* growth near drain inlets is critical for limiting environmental transmission of this bacterium.

Our experiments revealed the presence of a large number of Gram-negative non-fermenting bacteria in all eight drain samples. This group of bacteria includes *P. aeruginosa*, and is associated with waterborne transmission and nosocomial, opportunistic infections^20,21^. Overall, culturomics results suggest that the microbial compositions of our hospital’s drains largely consisted of *Acinetobacter*, *Pseudomonas*, *Sphingomonas*, and *Stenotrophomonas*; these findings are consistent with previous studies identifying these bacteria as common inhabitants of healthcare water systems^20–22^. However, we showed that the abundances of these four genera were generally lower in VIM-positive sink drain samples; the exception at species level was *P. aeruginosa*, which was present in all VIM-positive samples, and in extremely low abundance in VIM-negative samples. Some Gram-negative non-fermenting bacteria were completely absent in VIM-positive samples: *Acidovorax*, *Chryseobacterium*, *Flavobacterium*, and *Sphingobium.* A possible explanation for this could be active or passive competition between these non-fermenters and *P. aeruginosa* in this niche^23^. Surprisingly, certain species we anticipated finding based on their contributions to nosocomial infections were not found in any sample, namely *Klebsiella pneumoniae*, *Escherichia coli*, and *Staphylococcus aureus*.

In addition, two VIM-negative sink drain samples (Drains E^−^ and H^−^) and one VIM-positive sink drain sample with a low abundance of VIM-positive *P. aeruginosa* (Drain B^+^) contained *Candida* spp., especially *C. albicans* (Table 2). *P. aeruginosa* and *C. albicans* have previously been shown to competitively secrete metabolites that inhibit virulence or growth in one another^24^; such behavior may be occurring in sink environments, but this has not yet been reported. *C. albicans*, among other fungi, are also capable of modifying the pH of their environments by secreting organic acids^25^; interestingly, acetic acid has been used before to successfully decolonize hospital sinks harboring VIM-positive *P. aeruginosa*^26^. No study has yet investigated the ability of *P. aeruginosa* and *Candida* to coexist in hospital sinks, so it is of keen interest for us to determine if the presence or absence of *Candida*, as well as the aforementioned Gram-negative non-fermenting bacteria, inhibit colonization of VIM-positive *P. aeruginosa* in drains. It should be noted, however, that *Candida* are also capable of causing serious infections in hospitalized patients, and its presence in the innate hospital environment is worrisome as well. Nevertheless, microorganisms that produce acids may be useful probiotic candidates against environmental *P. aeruginosa*; besides *Candida*, the presences of *Lactobacillus* and *Acetobacter* in our negative drain samples (Drains E^−^ and F^−^, respectively) are of particular interest (Table 2). An increasing number of reports propose using *Bacillus* spp., such as *B. subtilis*, *B. megaterium*, and *B. pumilus*, as probiotic cleansers in hospitals; they do not produce acids, but have been shown to secrete an enzyme that disrupts the extracellular polymeric substance (EPS) matrix in clinically-relevant biofilms^27,28^. In our study, the same *Bacillus* spp*.* were found in some sinks, but in low loads; additionally, *Bacillus* were found in both positive and negative drain samples. While spores were specifically isolated using a thermic shock pre-culture condition, we did not microscopically confirm if *Bacillus* existed as spores in these samples. Therefore, it is possible that ungerminated *Bacillus* spores were missed by both the culture-dependent and culture-independent methods used. This is relevant, as *Bacillus* produce spores in response to unfavorable surroundings, and in one study, *B. subtilis* existed almost exclusively as spores in freshwater conditions^29^. The aforementioned probiotic sanitation method employed by Caselli et al. comprised a fixed concentration of *Bacillus* spores as well. Therefore, while *Bacillus* are less convincing as probiotic candidates for sinks based on our findings, we also cannot rule them out.

We also observed differences in culture media aimed at isolating *Pseudomonas* spp. (Table 3). Cetrimide agar is a culture medium selective for *Pseudomonas* spp., but *P. aeruginosa* was only detected on this medium in the two positive sink drain samples with the highest abundances of *P. aeruginosa* (Table 3). An in-house enrichment broth containing 2 mg/l ceftazidime and 50 mg/l vancomycin (CAZ-VAN) used by our laboratory for detecting VIM-positive *P. aeruginosa* was successful at detecting this microorganism in three of the positive sink drain samples, but not in the drain sample where *P. aeruginosa* was in lowest abundance (Drain D^+^) (Table 3). The most sensitive medium used to culture *P. aeruginosa* was Mueller Hinton (MH) agar made with tap water from the tested wards, which cultured *P. aeruginosa* from all four positive sink drain samples (Table 3). This medium was created to determine if compounds added to the tap water in these wards—20–50 µg/l silver and 400–600 µg/copper to inhibit *Legionella* growth—also affected the growth of *P. aeruginosa*. The success of this medium at detecting VIM-positive *P. aeruginosa* suggests that customizing culture conditions to mimic the environment of origin is an effective strategy for culturing this microorganism, even when in extremely low loads. It is also evident from Table 3 that this medium was successful at cultivating several genera from our drain samples, so in future culturomics studies targeting sinks, the number of culture conditions can be reduced when MH agar with tap water is used.

This study had some limitations. Firstly, we selected negative sinks based on our definition that VIM-positive *P. aeruginosa* had not been cultured from these sinks, even after exposure to patients carrying this bacterium; however, the exposure time and use of these sinks by culture-positive patients are unknown. Secondly, among the 179 culture conditions employed during culturomics, cell culture capable of isolating viruses and phages was not included. While viruses may have been present and influential on drain microbiota, and in light of the labor-intensiveness of culturomics, we decided to focus on cultivating bacteria and fungi only, as these are currently being used as probiotics. Thirdly, we based the purity of cultures on colony morphology, so some species may have gone unidentified if their morphologies were too similar in appearance to others. Fourthly, we did not measure biofilm-specific markers, such as those relevant to EPS formation, so it is unclear if biofilms were present on drain plugs at the moment they were removed; subtle differences in nutrient exposure and moisture between drain plugs, inlets, and siphons could have contributed to different biofilm compositions in these sites. Most importantly, uncultivable or difficult-to-culture microorganisms may have been missed, so culturomics cannot be considered a standalone method for microbiota analysis. This shortcoming can be overcome by using molecular techniques, such as 16S and 18S/ITS rRNA sequencing. One advantage of using culturomics over molecular techniques is that isolates are obtained in culture, and can be used for future analyses. Moreover, molecular methods are highly sensitive, and will amplify DNA of non-living microorganisms, providing no insight into the viability or interactions of microorganisms in a niche^30^. For these reasons, culturomics is a valuable addition to molecular methods for microbiota analysis, especially when the viability of microbiota is important.

In conclusion, the composition of VIM-positive *P. aeruginosa* reservoirs formed in hospital sink drains was until now an unexplored topic. We show that a high abundance of Gram-negative non-fermenting bacteria, including *Acinetobacter*, non-aeruginosa *Pseudomonas* spp., *Acidovorax*, *Chryseobacterium*, *Flavobacterium*, and *Sphingobium*, as well as *Candida* characterized sink drains where VIM-positive *P. aeruginosa* has never successfully formed environmental reservoirs. It is possible that these microorganisms inhibit persistence of *P. aeruginosa* by, for example, producing acids that prohibit long-term colonization. However, these results are a snapshot of the microbiota present at a single moment, and may produce different results if the same sink drains are re-cultured at a different moment, perhaps reflecting the microbiota of patients admitted to those rooms at the time. Furthermore, drain microbiota may be influenced by the frequency at which sinks are used, the composition of materials in the sinks and pipelines, exposure to nutrients and biocides, and other environmental factors, such as room temperature and humidity. Nevertheless, by examining positive and negative sink drains from different hospital rooms using a standardized culturomics approach, we were able to decipher patterns between environments where VIM-positive *P. aeruginosa* has been a successful colonizer, and environments where it has not, even though the number of included sink drain samples was low; the labor-intensiveness of experiments limited the number of sinks that we could analyze. Continuing research on sink drain composition should target a larger number of sinks, ideally from multiple hospitals, and combine culturomics with molecular methods. Additionally, the microbial consortia found should be tested in competition experiments, incorporating pH modulation, to draw more firm conclusions on which microorganisms enable or inhibit persistence of VIM-positive *P. aeruginosa* in sinks. These results may prove valuable in designing environmental probiotics that replace ineffective disinfectants to decolonize hospital sinks, or prevent formation of drain reservoirs. Due to increasing multidrug resistance and the prevalence of *P. aeruginosa* clone ST111 in hospitals, measures to prevent this microorganism from surviving in reservoirs, or to eradicate reservoirs that form, should be labeled high priority for infection prevention and control.

## Supplementary information

Supplementary Information.

## Data Availability

Isolates and datasets generated by this study are available on request from the corresponding author.
