# Supplementary material for: Novel use of culturomics to identify the microbiota in hospital sink drains with and without persistent VIM-positive Pseudomonas aeruginosa
Source: Sci Rep. 2020 Oct 13;10:17052. doi: 10.1038/s41598-020-73650-8 (PMC7554030; doi:10.1038/s41598-020-73650-8)
Supplement: Supplementary file 1 — Supplementary Information. [file 41598_2020_73650_MOESM1_ESM.pdf]

Novel use of culturomics to identify the microbiota in hospital sink drains with and without persistent VIM-positive *Pseudomonas aeruginosa*

Jannette Pirzadian[1], Susan P. Harteveld[1a], Shanice N. Ramdutt[1a], Willem J. B. van Wamel[1], Corné H. W. Klaassen[1], Margreet C. Vos[1], and Juliëtte A. Severin[1*]

Affiliation: [1]Department of Medical Microbiology and Infectious Diseases, Erasmus MC University Medical Center Rotterdam, Rotterdam, the Netherlands

[a]Authors contributed equally to this work.

***Corresponding author:** Dr. Juliëtte A. Severin, MD PhD, j.severin@erasmusmc.nl

Supplementary Figure S1: Designs and locations of sinks used in this study.

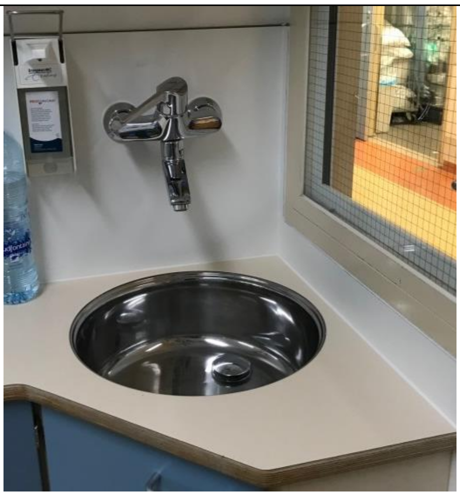

Drain A^{+}
Location: single-bed patient room; adult intensive care unit

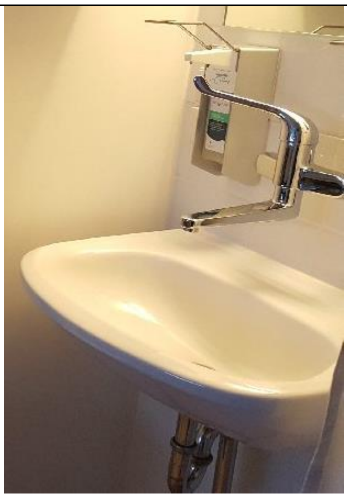

Drain B^{+}
Location: two-bed patient room; gastrointestinal surgery ward

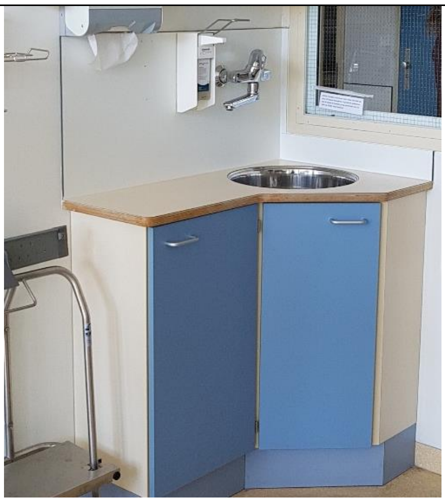

Drain C^{+}
Location: single-bed patient room; adult intensive care unit

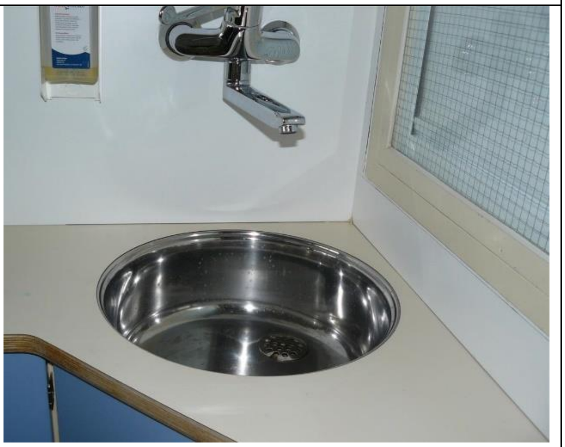

Drain D^{+}
Location: single-bed patient room; adult intensive care unit

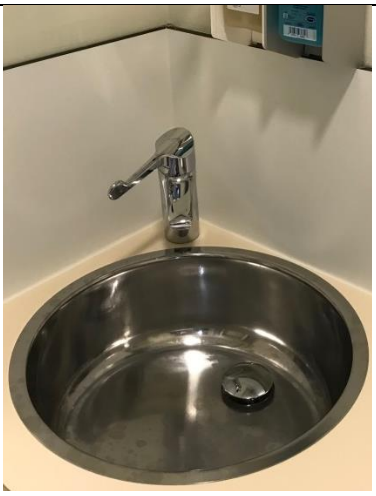<br>**Drain E^-**<br>Location: anteroom to a patient room; adult intensive care unit	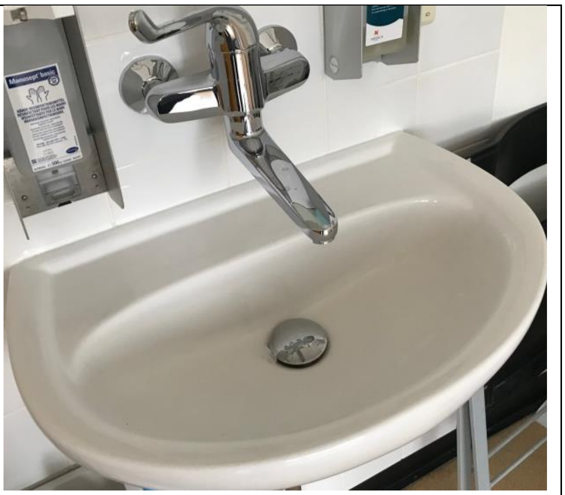<br>**Drain F^-**<br>Location: four-bed patient room; general surgery ward
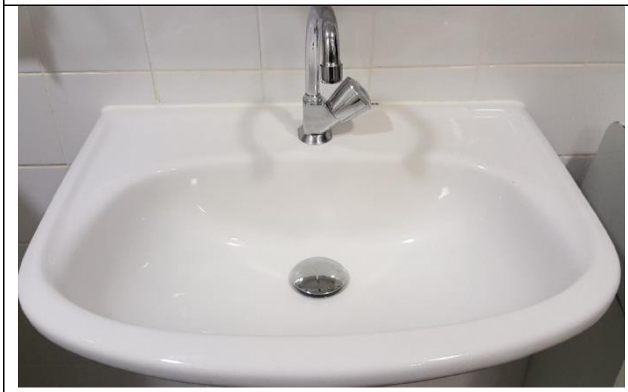<br>**Drain G^-**<br>Location: communal patient bathroom; gastrointestinal surgery ward	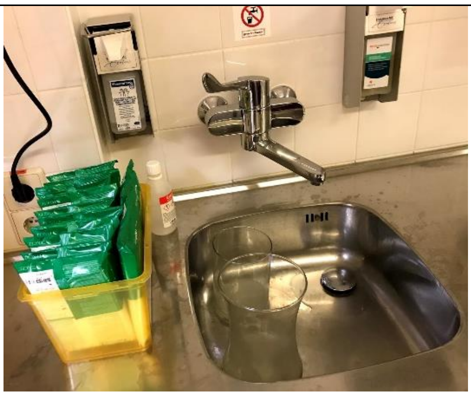<br>**Drain H^-**<br>Location: dirty utility room for healthcare workers; general surgery ward

Supplementary Fig. S1: Designs and locations of sinks used in this study. Drains A^+, B^+, C^+, and D^+ were sink drain samples containing VIM-positive *P. aeruginosa*. Drains E^-, F^-, G^-, and H^- were sink drain samples that did not contain VIM-positive *P. aeruginosa*. The name of the drain sample is given for each sink, as well as the sink's location regarding type of room and ward. In the image for Drain D^+, a drain trap no longer in use is shown. Drain plugs used in this study (visible in most images) were identical among the eight sinks. The image for Drain C^+ was taken by Tessa Souhoka and used with permission.

Supplementary Table S2: Complete list of culture conditions used in each culturomics experiment.

Bacteroides bile esculin agar, anaerobic, room temperature
Bacteroides bile esculin agar, anaerobic, 35°C
Blood culture preincubation, aerobic, room temperature
Blood culture preincubation, aerobic, 35°C
Blood culture preincubation, anaerobic, room temperature
Blood culture preincubation, anaerobic, 35°C
0.2 µm-sized pore prefiltration, blood culture preincubation, aerobic, room temperature
0.2 µm-sized pore prefiltration, blood culture preincubation, aerobic, 35°C
0.2 µm-sized pore prefiltration, blood culture preincubation, anaerobic, room temperature
0.2 µm-sized pore prefiltration, blood culture preincubation, anaerobic, 35°C
0.45 µm-sized pore prefiltration, blood culture preincubation, aerobic, room temperature
0.45 µm-sized pore prefiltration, blood culture preincubation, aerobic, 35°C
0.45 µm-sized pore prefiltration, blood culture preincubation, anaerobic, room temperature
0.45 µm-sized pore prefiltration, blood culture preincubation, anaerobic, 35°C
Thermic shock pretreatment, blood culture preincubation, aerobic, room temperature
Thermic shock pretreatment, blood culture preincubation, aerobic, 35°C
Thermic shock pretreatment, blood culture preincubation, anaerobic, room temperature
Thermic shock pretreatment, blood culture preincubation, anaerobic, 35°C
Brain heart infusion agar with 10 µg/ml kanamycin, aerobic, room temperature
Brain heart infusion agar with 10 µg/ml kanamycin, aerobic, 35°C
Brain heart infusion agar with 10 µg/ml kanamycin, aerobic with 5% CO_2, 35°C
Brain heart infusion agar with 10 µg/ml kanamycin, microaerophilic, room temperature
Brain heart infusion agar with 10 µg/ml kanamycin, microaerophilic, 35°C
Brain heart infusion agar with 10 µg/ml kanamycin, anaerobic, room temperature
Brain heart infusion agar with 10 µg/ml kanamycin, anaerobic, 35°C
Brain heart infusion agar with 10 µg/ml vancomycin, aerobic, room temperature
Brain heart infusion agar with 10 µg/ml vancomycin, aerobic, 35°C
Brain heart infusion agar with 10 µg/ml vancomycin, aerobic with 5% CO_2, 35°C
Brain heart infusion agar with 10 µg/ml vancomycin, microaerophilic, room temperature
Brain heart infusion agar with 10 µg/ml vancomycin, microaerophilic, 35°C
Brain heart infusion agar with 10 µg/ml vancomycin, anaerobic, room temperature
Brain heart infusion agar with 10 µg/ml vancomycin, anaerobic, 35°C
Brain heart infusion broth with 10 mg/l vitamin B6, aerobic, room temperature
Brain heart infusion broth with 10 mg/l vitamin B6, aerobic, 35°C
Brain heart infusion broth with 10 mg/l vitamin B6, aerobic with 5% CO_2, 35°C
Brucella agar with 5% sheep blood, aerobic, room temperature
Brucella agar with 5% sheep blood, aerobic, 35°C
Brucella agar with 5% sheep blood, aerobic with 5% CO_2, 35°C
Brucella agar with 5% sheep blood, microaerophilic, room temperature
Brucella agar with 5% sheep blood, microaerophilic, 35°C
Brucella agar with 5% sheep blood, anaerobic, room temperature
Brucella agar with 5% sheep blood, anaerobic, 35°C
0.2 µm-sized pore prefiltration, Brucella agar with 5% sheep blood, anaerobic, room temperature
0.2 µm-sized pore prefiltration, Brucella agar with 5% sheep blood, anaerobic, 35°C

0.45 µm-sized pore prefiltration, Brucella agar with 5% sheep blood, anaerobic, room temperature
0.45 µm-sized pore prefiltration, Brucella agar with 5% sheep blood, anaerobic, 35°C
Thermic shock pretreatment, Brucella agar with 5% sheep blood, anaerobic, room temperature
Thermic shock pretreatment, Brucella agar with 5% sheep blood, anaerobic, 35°C
Burkholderia cepacia selective agar, aerobic, room temperature
Burkholderia cepacia selective agar, aerobic, 35°C
Burkholderia cepacia selective agar, aerobic with 5% CO2, 35°C
Burkholderia cepacia selective agar, microaerophilic, room temperature
Burkholderia cepacia selective agar, microaerophilic, 35°C
Centers for Disease Control and Prevention (CDC) anaerobe 5% sheep blood agar with phenylethyl alcohol, anaerobic, room temperature
Centers for Disease Control and Prevention (CDC) anaerobe 5% sheep blood agar with phenylethyl alcohol, anaerobic, 35°C
Cetrimide agar, aerobic, room temperature
Cetrimide agar, aerobic, 35°C
Cetrimide agar, aerobic with 5% CO2, 35°C
Cetrimide agar, microaerophilic, room temperature
Cetrimide agar, microaerophilic, 35°C
Cetrimide agar, anaerobic, room temperature
Cetrimide agar, anaerobic, 35°C
Chocolate agar, aerobic, room temperature
Chocolate agar, aerobic, 35°C
Chocolate agar, aerobic with 5% CO2, 35°C
Chocolate agar, microaerophilic, room temperature
Chocolate agar, microaerophilic, 35°C
Chocolate agar, anaerobic, room temperature
Chocolate agar, anaerobic, 35°C
Eosin methylene blue agar, aerobic, room temperature
Eosin methylene blue agar, aerobic, 35°C
Eosin methylene blue agar, aerobic with 5% CO2, 35°C
Eosin methylene blue agar, microaerophilic, room temperature
Eosin methylene blue agar, microaerophilic, 35°C
Extended spectrum beta-lactamase (ESBL) agar, aerobic, room temperature
Extended spectrum beta-lactamase (ESBL) agar, aerobic, 35°C
Extended spectrum beta-lactamase (ESBL) agar, aerobic with 5% CO2, 35°C
Extended spectrum beta-lactamase (ESBL) agar, microaerophilic, room temperature
Extended spectrum beta-lactamase (ESBL) agar, microaerophilic, 35°C
Extended spectrum beta-lactamase (ESBL) agar, anaerobic, room temperature
Extended spectrum beta-lactamase (ESBL) agar, anaerobic, 35°C
Gonococcal (GC) agar with supplement VX and 2% hemoglobin, aerobic, room temperature
Gonococcal (GC) agar with supplement VX and 2% hemoglobin, aerobic, 35°C
Gonococcal (GC) agar with supplement VX and 2% hemoglobin, aerobic with 5% CO2, 35°C
Gonococcal (GC) agar with supplement VX and 2% hemoglobin, microaerophilic, room temperature
Gonococcal (GC) agar with supplement VX and 2% hemoglobin, microaerophilic, 35°C
Hektoen enteric agar, aerobic, room temperature

Hektoen enteric agar, aerobic, 35°C
Hektoen enteric agar, aerobic with 5% CO_2, 35°C
Hektoen enteric agar, microaerophilic, room temperature
Hektoen enteric agar, microaerophilic, 35°C
Legionella buffered charcoal yeast extract agar with 0.4 g/l L-cysteine hydrochloride, aerobic, room temperature
Legionella buffered charcoal yeast extract agar with 0.4 g/l L-cysteine hydrochloride, aerobic, 35°C
Legionella buffered charcoal yeast extract agar with 0.4 g/l L-cysteine hydrochloride, microaerophilic, room temperature
Legionella buffered charcoal yeast extract agar with 0.4 g/l L-cysteine hydrochloride, microaerophilic, 35°C
Legionella buffered charcoal yeast extract agar with antibiotics, aerobic, room temperature
Legionella buffered charcoal yeast extract agar with antibiotics, aerobic, 35°C
Legionella buffered charcoal yeast extract agar with antibiotics, microaerophilic, room temperature
Legionella buffered charcoal yeast extract agar with antibiotics, microaerophilic, 35°C
MacConkey agar, aerobic, room temperature
MacConkey agar, aerobic, 35°C
MacConkey agar, aerobic with 5% CO_2, 35°C
MacConkey agar, microaerophilic, room temperature
MacConkey agar, microaerophilic, 35°C
MacConkey agar, anaerobic, room temperature
MacConkey agar, anaerobic, 35°C
Middlebrook 7H10 agar, aerobic, room temperature
Middlebrook 7H10 agar, aerobic, 35°C
Middlebrook 7H10 agar, aerobic with 5% CO_2, 35°C
Middlebrook 7H10 agar, microaerophilic, room temperature
Middlebrook 7H10 agar, microaerophilic, 35°C
Mueller Hinton agar with 10 µg/ml kanamycin, aerobic, room temperature
Mueller Hinton agar with 10 µg/ml kanamycin, aerobic, 35°C
Mueller Hinton agar with 10 µg/ml kanamycin, aerobic with 5% CO_2, 35°C
Mueller Hinton agar with 10 µg/ml kanamycin, microaerophilic, room temperature
Mueller Hinton agar with 10 µg/ml kanamycin, microaerophilic, 35°C
Mueller Hinton agar with 10 µg/ml kanamycin, anaerobic, room temperature
Mueller Hinton agar with 10 µg/ml kanamycin, anaerobic, 35°C
Mueller Hinton agar with 10 µg/ml vancomycin, aerobic, room temperature
Mueller Hinton agar with 10 µg/ml vancomycin, aerobic, 35°C
Mueller Hinton agar with 10 µg/ml vancomycin, aerobic with 5% CO_2, 35°C
Mueller Hinton agar with 10 µg/ml vancomycin, microaerophilic, room temperature
Mueller Hinton agar with 10 µg/ml vancomycin, microaerophilic, 35°C
Mueller Hinton agar with 10 µg/ml vancomycin, anaerobic, room temperature
Mueller Hinton agar with 10 µg/ml vancomycin, anaerobic, 35°C
Mueller Hinton agar with 5% sheep blood, aerobic, room temperature
Mueller Hinton agar with 5% sheep blood, aerobic, 35°C
Mueller Hinton agar with 5% sheep blood, aerobic with 5% CO_2, 35°C
Mueller Hinton agar with 5% sheep blood, microaerophilic, room temperature
Mueller Hinton agar with 5% sheep blood, microaerophilic, 35°C
Mueller Hinton agar with 5% sheep blood, anaerobic, room temperature
Mueller Hinton agar with 5% sheep blood, anaerobic, 35°C

Mueller Hinton agar with hospital tap water, aerobic, room temperature
Mueller Hinton agar with hospital tap water, aerobic, 35°C
Mueller Hinton agar with hospital tap water, aerobic with 5% CO_2, 35°C
Mueller Hinton agar with hospital tap water, microaerophilic, room temperature
Mueller Hinton agar with hospital tap water, microaerophilic, 35°C
Mueller Hinton agar with hospital tap water, anaerobic, room temperature
Mueller Hinton agar with hospital tap water, anaerobic, 35°C
Phenyl mannitol agar with 75 g/l sodium chloride, aerobic, room temperature
Phenyl mannitol agar 75 g/l sodium chloride, aerobic, 35°C
Phenyl mannitol agar with 75 g/l sodium chloride, aerobic with 5% CO_2, 35°C
Phenyl mannitol agar with 75 g/l sodium chloride, microaerophilic, room temperature
Phenyl mannitol agar with 75 g/l sodium chloride, microaerophilic, 35°C
Reasoner's 2A agar, aerobic, room temperature
Reasoner's 2A agar, aerobic, 35°C
Reasoner's 2A agar, aerobic with 5% CO_2, 35°C
Reasoner's 2A agar, microaerophilic, room temperature
Reasoner's 2A agar, microaerophilic, 35°C
Tryptic soy agar with 5% sheep blood (TSA-SB), aerobic, room temperature
Tryptic soy agar with 5% sheep blood (TSA-SB), aerobic, 35°C
Tryptic soy agar with 5% sheep blood (TSA-SB), aerobic with 5% CO_2, 35°C
Tryptic soy agar with 5% sheep blood (TSA-SB), microaerophilic, room temperature
Tryptic soy agar with 5% sheep blood (TSA-SB), microaerophilic, 35°C
Tryptic soy agar with 5% sheep blood (TSA-SB), anaerobic, room temperature
Tryptic soy agar with 5% sheep blood (TSA-SB), anaerobic, 35°C
0.2 µm-sized pore prefiltration, TSA-SB, aerobic, room temperature
0.2 µm-sized pore prefiltration, TSA-SB, aerobic, 35°C
0.2 µm-sized pore prefiltration, TSA-SB, microaerophilic, room temperature
0.2 µm-sized pore prefiltration, TSA-SB, microaerophilic, 35°C
0.2 µm-sized pore prefiltration, TSA-SB, anaerobic, room temperature
0.2 µm-sized pore prefiltration, TSA-SB, anaerobic, 35°C
0.45 µm-sized pore prefiltration, TSA-SB, aerobic, room temperature
0.45 µm-sized pore prefiltration, TSA-SB, aerobic, 35°C
0.45 µm-sized pore prefiltration, TSA-SB, microaerophilic, room temperature
0.45 µm-sized pore prefiltration, TSA-SB, microaerophilic, 35°C
0.45 µm-sized pore prefiltration, TSA-SB, anaerobic, room temperature
0.45 µm-sized pore prefiltration, TSA-SB, anaerobic, 35°C
Thermic shock pretreatment, TSA-SB, aerobic, room temperature
Thermic shock pretreatment, TSA-SB, aerobic, 35°C
Thermic shock pretreatment, TSA-SB, microaerophilic, room temperature
Thermic shock pretreatment, TSA-SB, microaerophilic, 35°C
Thermic shock pretreatment, TSA-SB, anaerobic, room temperature
Thermic shock pretreatment, TSA-SB, anaerobic, 35°C
Tryptic soy broth with 2 mg/l ceftazidime and 50 mg/l vancomycin, aerobic, room temperature (CAZ-VAN enrichment broth)
Tryptic soy broth with 2 mg/l ceftazidime and 50 mg/l vancomycin, aerobic, 35°C (CAZ-VAN enrichment broth)
Tryptic soy broth with 2 mg/l ceftazidime and 50 mg/l vancomycin, aerobic with 5% CO_2, 35°C (CAZ-VAN enrichment broth)
Tryptic soy broth with 4 mg/l gentamycin and 1 mg/l norfloxacin, aerobic, room temperature

Tryptic soy broth with 4 mg/l gentamycin and 1 mg/l norfloxacin, aerobic, 35°C
Tryptic soy broth with 4 mg/l gentamycin and 1 mg/l norfloxacin, aerobic with 5% CO2, 35°C
Tryptic soy broth with 4 mg/l tobramycin and 1 mg/l norfloxacin, aerobic, room temperature
Tryptic soy broth with 4 mg/l tobramycin and 1 mg/l norfloxacin, aerobic, 35°C
Tryptic soy broth with 4 mg/l tobramycin and 1 mg/l norfloxacin, aerobic with 5% CO2, 35°C
No sonication (drain plug sampled using a sterile scalpel), TSA-SB, aerobic, 35°C
No sonication (drain plug sampled using a sterile scalpel), TSA-SB, aerobic with 5% CO2, 35°C
No sonication (drain plug sampled using a sterile scalpel), TSA-SB, microaerophilic, 35°C
No sonication (drain plug sampled using a sterile scalpel), TSA-SB, anaerobic, 35°C
5 minutes sonication, TSA-SB, aerobic, 35°C
5 minutes sonication, Brucella agar with 5% sheep blood, aerobic, 35°C
5 minutes sonication, TSA-SB, anaerobic, 35°C
5 minutes sonication, Brucella agar with 5% sheep blood, anaerobic, 35°C

Supplementary Table S2: Complete list of culture conditions used in each culturomics experiment. Unless “no sonication” is indicated, all culture conditions were used following 1 minute sonication; the exception is “5 minutes sonication,” in which culture conditions were used following 5 cumulative minutes sonication.

Supplementary Table S3: Identifiable species within each genus, and their most optimal tested culture conditions.

Genus	Species	Most optimal tested culture conditions
Acetobacter	*A. indonesiensis*	n.a.
Acidovorax	*A. temperans*	Non-selective media; 35°C; aerobic conditions
Acinetobacter	*A. haemolyticus*<br>*A. johnsonii*<br>*A. parvus*<br>*A. ursingii*	Media selective for Gram-negative bacteria (incl. vancomycin); 35°C or room temperature; aerobic or microaerophilic conditions
Actinomyces	*A. neuii*	n.a.
Aspergillus	*A. fumigatus*<br>*A. hiratsukae*<br>*A. versicolor*	Non-selective media; 35°C or room temperature; microaerophilic conditions
Bacillus	*B. cereus*<br>*B. circulans*<br>*B. clausii*<br>*B. flexus*<br>*B. halosaccharovorans*<br>*B. infantis*<br>*B. licheniformis*<br>*B. megaterium*<br>*B. pumilus*<br>*B. simplex*	Thermic shock pretreatment; non-selective media; 35°C or room temperature; aerobic or microaerophilic conditions
Brevibacillus	*B. parabrevis*	n.a.
Brevibacterium	*B. casei*	Media selective for Gram-positive bacteria (incl. kanamycin); 35°C; aerobic conditions
Brevundimonas	*B. diminuta*	Non-selective media; room temperature; aerobic conditions
Candida	*C. albicans*<br>*C. guilliermondii*<br>*C. lusitaniae*	Non-selective media or phenyl mannitol agar + 75 g/l sodium chloride; 35°C or room temperature; aerobic or microaerophilic conditions
Chryseobacterium	*C. scophthalmum*<br>*C. ureilyticum*	Non-selective media; room temperature; aerobic or microaerophilic conditions
Citrobacter	*C. freundii*	Media selective for Gram-negative bacteria (incl. vancomycin) or ESBL agar; 35°C or room temperature; aerobic or microaerophilic conditions
Clostridium	*C. algidixylanolyticum*	n.a.
Cutibacterium	*C. acnes*	Non-selective media; 35°C; anaerobic conditions
Delftia	*D. acidovorans*	Media selective for Gram-negative bacteria; 35°C or room temperature; aerobic or microaerophilic conditions
Dietzia	*D. cinnamea*	n.a.

Enterobacter	*E. asburiae*<br>*E. cloacae*<br>*E. kobei*	Media selective for Gram-negative bacteria or ESBL agar; 35°C or room temperature; microaerophilic or anaerobic conditions
Enterococcus	*E. faecalis*	Non-selective media; 35°C or room temperature; microaerophilic or anaerobic conditions
Facklamia	*F. hominis*	n.a.
Finegoldia	*F. magna*	n.a.
Flavobacterium	*F. oncorhynchi*<br>*F. plurextorum*<br>*F. tructae*	Non-selective media; room temperature; aerobic or microaerophilic conditions
Geotrichum	*G. candidum*<br>*G. silvicola*	Non-selective media; room temperature; microaerophilic conditions
Kocuria	*K. marina*<br>*K. rhizophila*	Non-selective media; 35°C or room temperature; aerobic or microaerophilic conditions
Lactobacillus	*L. paracasei*<br>*L. rhamnosus*	Non-selective media; 35°C or room temperature; microaerophilic or anaerobic conditions
Micrococcus	*M. luteus*	Non-selective media; 35°C or room temperature; aerobic or microaerophilic conditions
Moraxella	*M. osloensis*	n.a.
Ochrobactrum	*O. anthropi*	n.a.
Paenibacillus	*P. glucanolyticus*	n.a.
Pseudomonas	*P. aeruginosa*<br>*P. brenneri*<br>*P. gessardii*<br>*P. koreensis*<br>*P. monteilii*<br>*P. oleovorans*<br>*P. proteolytica*<br>*P. pseudoalcaligenes*<br>*P. putida*<br>*P. rhodesiae*	Non-selective media (incl. tap water), ESBL agar, cetrimide agar, or CAZ-VAN enrichment broth; 35°C or room temperature; aerobic or microaerophilic conditions
Pseudoxanthomonas	*P. mexicana*	n.a.
Roseomonas	*R. mucosa*	n.a.
Rothia	*R. amarae*	n.a.
Sphingobacterium	*S. spiritivorum*	n.a.
Sphingobium	*S. yanoikuyae*	Non-selective media; 35°C or room temperature; aerobic or microaerophilic conditions
Sphingomonas	*S. adhaesiva*<br>*S. aerolata*<br>*S. faeni*<br>*S. panni*<br>*S. paucimobilis*	Non-selective media; 35°C or room temperature; aerobic or microaerophilic conditions

Staphylococcus	*S. capitis*<br>*S. epidermidis*<br>*S. haemolyticus*<br>*S. hominis*<br>*S. saprophyticus*<br>*S. warneri*	Non-selective media or phenyl mannitol agar + 75 g/l sodium chloride; 35°C; aerobic or microaerophilic conditions
Stenotrophomonas	*S. maltophilia*	Media selective for Gram-negative bacteria (incl. vancomycin); 35°C or room temperature; aerobic or microaerophilic conditions
Streptococcus	*S. mutans*<br>*S. sanguinis*<br>*S. sobrinus*	Non-selective media; 35°C; microaerophilic or anaerobic conditions
Trichosporon	*T. asahii*	Non-selective media or phenyl mannitol agar + 75 g/l sodium chloride; 35°C; aerobic or microaerophilic conditions

Supplementary Table S3: Identifiable species within each genus, and their most optimal tested culture conditions. Shown are all discernible species identified by MALDI-TOF and/or 16S rRNA sequencing, and their most optimal culture conditions based on culturomics findings. Culture conditions are not exhaustive, but suggest those conditions that were the most successful at isolating the given genus/species in culture during our study. Genera that could not be identified to species level are not shown. n.a.=the species grew infrequently, or in a limited number of culture conditions.
